# Supplementary material for: Statistical learning goes beyond the d-band model providing the thermochemistry of adsorbates on transition metals
Source: Nat Commun. 2019 Oct 15;10:4687. doi: 10.1038/s41467-019-12709-1 (PMC6794282; doi:10.1038/s41467-019-12709-1)
Supplement: Supplementary file 1 — Supplementary Information [file 41467_2019_12709_MOESM1_ESM.pdf]

Supplementary information

Statistical learning goes beyond the *d*-band model providing the thermochemistry of adsorbates on transition metals

García-Muelas *et al.*

Supplementary Methods

Principal Component Analysis (PCA) algorithm

The PCA algorithm, summarized in Figure 1 and Supplementary Figure 3, was applied as follows:

1. Calculate the formation energy for the species on each metal, $E_{\mathrm{C_xH_yO_z}*}$, by DFT and Supplementary Equations 1-2. Group the energies into matrix $\mathbf{E} = (E_{ij})$. Each row i corresponds to a metal and each column j to a species. For m species and n metals, $\mathbf{E}$ has size $n \times m$. The elements of $\mathbf{E}$ are in eV.

$$x\mathrm{CH_4} + \left(-2x + \tfrac{1}{2}y - z\right)\mathrm{H_2} + z\mathrm{H_2O} +^{*} \longrightarrow \mathrm{C}_x\mathrm{H}_y\mathrm{O}_z^{*} \tag{1}$$

$$E_{\mathrm{C}_x\mathrm{H}_y\mathrm{O}_z^{*}} = E^{\mathrm{VASP}}_{\mathrm{C}_x\mathrm{H}_y\mathrm{O}_z^{*}} - xE^{\mathrm{VASP}}_{\mathrm{CH_4}} + \left(2x - \tfrac{1}{2}y + z\right)E^{\mathrm{VASP}}_{\mathrm{H_2}} - zE^{\mathrm{VASP}}_{\mathrm{H_2O}} - E^{\mathrm{VASP}}_{*} \tag{2}$$

2. Calculate the average μ_j (in eV) from Supplementary Equation 3 for each column of $\mathbf{E}$.

$$\mu_j = \frac{1}{n}\sum_{i=1}^{n} E_{ij} \tag{3}$$

3. Center $\mathbf{E}$ to get $\mathbf{X}$ from Supplementary Equation 4. It is not necessary to normalize $\mathbf{E}$ by including standard deviations.[1,2] $\mathbf{X}$ has size $n \times m$ and its elements are in eV.

$$x_{ij} = E_{ij} - \mu_j \tag{4}$$

   For PCA, standard deviations are commonly included to normalize input matrices.[1,2] However, our input matrix $\mathbf{E}$ contains only thermochemical data in consistent units, eV. Thus, including stardard deviations gives more weight to molecules whose adsorption energy is rather constant, such as $\mathrm{CH_4}$, and lower the prediction accuracy of larger molecules with higher number of unsaturations.

4. Calculate the covariance matrix, $\mathbf{C}$, from Supplementary Equation 5. The size of $\mathbf{C}$ is $m \times m$ and its elements are in eV^2.

$$\mathbf{C} = \mathbf{X}^t\mathbf{X} \tag{5}$$

5. Diagonalize $\mathbf{C}$ as a symmetric matrix, Supplementary Equation 6. The matrix $\mathbf{D}$ contains the eigenvalues of $\mathbf{C}$ in descending order. $\mathbf{V}$ is the matrix of eigenvectors associated to $\mathbf{D}$. $\mathbf{D}$ and $\mathbf{V}$ have the same size as $\mathbf{C}$, $m \times m$. The elements of $\mathbf{D}$ are in eV^2 and the ones of $\mathbf{V}$ are dimensionless.

$$\mathbf{D} = \mathbf{V}^t\mathbf{C}\mathbf{V} \tag{6}$$

6. Select the number of principal components, $k_{\max}$.

7. Generate the matrix $\mathbf{W}$ taking the first $k_{\max}$ columns of $\mathbf{V}$. $\mathbf{W}$ has size $m \times k_{\max}$.

8. Project $\mathbf{X}$ onto the basis vectors contained in the columns of $\mathbf{W}$, to get matrix $\mathbf{T}$. $\mathbf{T}$ has size $n \times k_{\max}$ and its components are in eV.

$$\mathbf{T} = \mathbf{XW} \tag{7}$$

9. The values of $\mathbf{X}$ can be estimated from Supplementary Equation 8 taking $k_{\max}$ principal components. Get the formation energy for molecule j on metal i, E_{ij} by undoing the centering step, Equations 4 and 9.

$$\hat{\mathbf{X}} = \mathbf{TW}^t \tag{8}$$

$$\hat{E}_{ij} = t_{i1}w_{1j} + t_{i2}w_{2j} + \cdots + t_{ik}w_{kj} + \cdots + t_{ik_{\max}}w_{k_{\max}j} + \mu_j \tag{9}$$

10. Increase the number of principal components, $k_{\max}$, until reaching the desired accuracy. To this end, two criteria were used in this study:[1]

    - Compare the accumulated variance for different values of $k_{\max}$. This can be obtained by adding up the $k_{\max}$ first elements of the diagonal matrix $\mathbf{D}$. A percentage of the variance can be obtaining by dividing that number by the trace of $\mathbf{D}$. In this study we used 95% of the total variance as threshold.
    - Compare the maximum, minimum, mean average, and standard errors for different values of $k_{\max}$ until the error reduction stagnates. At that point, increasing $k_{\max}$ may lead to overfitting.[1]

Selection of representative predictor molecules

To provide a rapid survey on the adsorption energies of adsorbates on a given alloy, it is desirable to calculate by DFT only a small subset of adsorbates, called predictors in the main text. Their number should be at least equal to the number of principal components, $k_{\max}$. However, even small variations in their energies may introduce a large noise in the thermochemistry being estimated. This noise can be reduced by taking more predictors than principal components and then following a Principal Component Regression. In case the number of principal components were equal to the number of predictors, the results would be analogous to the reported in Ref. [3,4] The representative predictors were obtained following these steps:

1. Apply a principal component analysis. Obtain the eigenvalues and eigenvectors matrices, $\mathbf{D}$ and $\mathbf{V}$, and determine how many principal components should be used.

2. Compare the predicted and DFT formation energies for all the species to get the prediction errors ε_j by Supplementary Equation 10 and calculate the standard error for the predictions σ_j Supplementary Equation 11. The species with high

standard error within the selected principal components are not good predictors as they increase the systematic error when estimating the thermochemistry of other adsorbates.

$$\varepsilon_j = \hat{E}_{C_xH_yO_z^*} - E_{C_xH_yO_z^*} \tag{10}$$

$$\sigma_j = \sqrt{\frac{1}{m}\sum_j^m \varepsilon_j^2} \tag{11}$$

3. Obtain the robustness for species j for becoming a predictor, ι_j, using Supplementary Equation 12. There, λ_k is the k-th eigenvalues of matrix $\mathbf{C}$, in eV^2, w_{kj} is the k-th descriptor for the molecule j. By definition, ι_j is a dimensionless, real positive number, although in the present study it took values between 8 and 83.

$$\iota_j = \frac{+\sqrt{\lambda_1 w_{1j}^2 + \lambda_2 w_{2j}^2 + \cdots + \lambda_l w_{k_{\max}j}^2}}{\sigma_j} \tag{12}$$

4. Select a set of predictors. Give preferences to the ones that have a high ι_j, and whose descriptors w_{kj} expands a large area (i.e., they are orthogonal).

Principal Component Regression (PCR) algorithm

For PCR, the metals are divided into training and validation (prediction) sets. In the training set, the formation energies of all molecules needs to be known. In the validation (prediction) set, only the formation energies of the predictors are needed. The PCR was applied in two ways: i) To validate the PCA results by a Leave-One-Out (L1O) test, and ii) To estimate the formation energies of all species on single-atom and near-surface alloys. The procedure, summarized in Figure 1 and Supplementary Figure 3, was applied as follows:

1. Split the energies in the training set into a matrix containing only the predictors, $\mathbf{E}'$, and another containing the rest of the species, $\mathbf{E}''$. In the main text, this matrix contained 3 predictors (O*, OH*, and CCHOH*) on 12 metals. For PCR-L1O, only 11 metals were taken.

2. Center $\mathbf{E}'$ to get $\mathbf{X}'$, Supplementary Equation 4. It is not necessary to center and $\mathbf{E}''$.

3. Apply a Principal Component Analysis on $\mathbf{X}'$ to obtain the matrices $\mathbf{D}'$ (eigenvalues) and $\mathbf{V}'$ (eigenvectors).

4. Take the first $k_{\max}$ columns of $\mathbf{V}'$ to get $\mathbf{W}'$, which is associated only to the predictors.

5. For each metal in the validation (prediction) set, put the energies of the predictors into the row matrix $\mathbf{E}_{\text{val}}$. Center $\mathbf{E}_{\text{val}}$ using the averages from matrix $\mathbf{E}'$ to get $\mathbf{X}_{\text{val}}$

6. Project $\mathbf{X}_{\text{val}}$ onto the basis vectors contained in the columns of $\mathbf{W}'$, to get the row matrix $\mathbf{T}'$, Supplementary Equation 13. $\mathbf{T}'$ has size $1 \times k_{\text{max}}$ and its components are in eV.

$$\mathbf{T}_{\text{val}} = \mathbf{X}_{\text{val}}\mathbf{W}' \tag{13}$$

7. Approximate the descriptors for the species in the validation (prediction) set, $\{w_{kj,\text{val}}\}$ and $\mu_{j,\text{val}}$, by a linear regression on Supplementary Equation 14.

$$E''_{ij} = t'_{i1}w_{1j,\text{val}} + t'_{i2}w_{2j,\text{val}} + \cdots + t'_{ik_{\text{max}}}w_{k_{\text{max}}j,\text{val}} + \mu_{j,\text{val}} \tag{14}$$

8. Finally, the energies from the validation (prediction) set can be estimated from Supplementary Equation 15.

$$\hat{E}_{ij,\text{val}} = t_{i1,\text{val}}w_{1j,\text{val}} + t_{i2,\text{val}}w_{2j,\text{val}} + \cdots + t_{ik_{\text{max}},\text{val}}w_{k_{\text{max}}j,\text{val}} + \mu_{j,\text{val}} \tag{15}$$

Generation of near-surface and single-atom alloys

1. As starting point, take the supercell corresponding to the host metal.

2. Substitute the top layer or the second-to-top layer by the guest metal to generate overlayers (`ol`) and subsurface (`ss`) alloys.[5] For single-atom alloys (`sa`), only the central atom on the topmost layer was replaced by the guest metal. There were 15 guest metals: Fe, Co, Re, and the 12 metals from the training set. Originally, there were 168 ($12 * (15 - 1)$) alloys for each type.

3. Adsorb the predictors.

4. Relax the structures generated in the two previous steps by DFT.

5. After applying this procedure, a total of 165 single-atom, 122 overlayers, and 156 subsurface alloys converged to the desired structures. Those alloys that contained any unconverged structure were removed from the pool:

   Single-atom alloys (SAA): Re/Au, Re/Zn, Re/Pt

   Overlayers (NSA-OL): Co/Ag, Cu/Ag, Fe/Ag, Ir/Ag, Ni/Ag, Os/Ag, Re/Ag, Rh/Ag, Ru/Ag, Co/Au, Fe/Au, Ir/Au, Ni/Au, Os/Au, Re/Au, Rh/Au, Ru/Au, Co/Cd, Cu/Cd, Fe/Cd, Ir/Cd, Ni/Cd, Os/Cd, Pt/Cd, Re/Cd, Rh/Cd, Ru/Cd, Zn/Cd, Ag/Cu, Au/Cu, Cd/Cu, Re/Cu, Cd/Ir, Ag/Ni, Au/Ni, Cd/Ni, Ir/Ni, Os/Ni, Pt/Ni, Re/Ni, Rh/Ni, Ru/Ni, Cd/Os, Cd/Rh, Cd/Ru, Cd/Zn.

   Subsurface (NSA-SS): Re/Cd, Fe/Cd, Au/Cu, Cd/Cu, Au/Ni, Cd/Ni, Ir/Ni, Os/Ni, Re/Ni, Ag/Os, Au/Os, Cd/Os.

Getting data from ioChem-BD

1. Login to the desired collection. The link for the present article is:

   https://doi.org/10.19061/iochem-bd-1-43

2. A tutorial is available in the first visit.

3. Click on the title of any calculation to see its structure, energy, and other related metadata.

   - For adsorbed C_0-C_2 species on clean metals, tags have the format `MM-XXXX`, where `Mm` is the metal: Cu, Ag, Au, etc., and `XXXX` is a code shown in Ref. [6]. The description includes the formula of all intermediates.
   - For single-atom alloys, (`AA`=`sa`), overlayers (`AA`=`ol`), and subsurface alloys (`AA`=`ss`), the tags have the format `AA-M1M2-XXXX`. `M1` and `M2` corresponds to the host and guest metals respectively.

4. To download a `.csv` file containing the full set of energies with their tags, click on "`Export .csv`" on the top right corner.

5. The `.csv` file should be opened using the `UTF-8` encoding.

   - In LibreOffice Calc, `UTF-8` is the default encoding. Use commas as delimiters when importing the data. No further actions are requested.
   - In Microsoft Excel: Open a new spreadsheet, click on "Data" menu, "From Text" button, select the `.csv` file, on "File Origin" choose "65001 Unicode UTF-8", and use commas as delimiters.

Supplementary Tables

Supplementary Table 1: Structural and van der Waals parameters for metals. DFT and experimental[7] lattice parameters (a, in Å) for *fcc* and *hcp* metals in this study. $[\frac{c}{a}]$ ratio is included for *hcp* metals. R_0 and C_6 (in Å and J nm^6 mol^{-1}) parameters for the Grimme's D2 method[8] were obtained following the procedure on Ref. [9]. The lattice parameters were obtained by regression using a linearized form of the Birch Murnaghan equation. Both a_{DFT} and $[\frac{c}{a}]_{\mathrm{DFT}}$ were converged to 10^{-5} Å.

Metal	a_{DFT}	$[\frac{c}{a}]_{\mathrm{DFT}}$	a_{EXP}	$[\frac{c}{a}]_{\mathrm{EXP}}$	R_0	C_6
Cu	3.634	–	3.615	–	1.562	2.740
Ag	4.147	–	4.086	–	1.819	5.481
Au	4.156	–	4.079	–	1.823	7.308
Ni	3.518	–	3.524	–	1.543	2.383
Pd	3.939	–	3.890	–	1.690	5.510
Pt	3.968	–	3.924	–	1.750	7.000
Rh	3.824	–	3.803	–	1.677	4.364
Ir	3.872	–	3.839	–	1.698	6.163
Ru	2.710	1.581	2.706	1.582	1.639	4.168
Os	2.740	1.601	2.734	1.606	1.504	5.878
Zn	2.715	1.734	2.665	1.856	1.531	3.419
Cd	3.031	1.907	2.979	1.886	1.764	5.219

DFT values were obtained from VASP,[10,11] using the PBE functional,[12] PAW pseudopotentials,[13,14] and a $15\times15\times15$ k-point mesh.

Supplementary Table 2: Electronic parameters for metals. Calculated d-band center referred to the Fermi level, in eV, and experimental[7] reduction potentials, in V, for the metals under study.

Metal	$\varepsilon_d - \varepsilon_F$	Reaction	E_h
Cu	–2.41	$Cu^{2+} + 2\,e^- \rightleftharpoons Cu$	+0.342
Ag	–4.09	$Ag^{+} + e^- \rightleftharpoons Ag$	+0.800
Au	–3.48	$Au^{+} + e^- \rightleftharpoons Au$	+1.692
Ni	–1.32	$Ni^{2+} + 2\,e^- \rightleftharpoons Ni$	−0.257
Pd	–1.74	$Pd^{2+} + 2\,e^- \rightleftharpoons Pd$	+0.951
Pt	–2.24	$Pt^{2+} + 2\,e^- \rightleftharpoons Pt$	+1.180
Rh	–1.83	$Rh^{+} + e^- \rightleftharpoons Rh$	+0.600
Ir	–1.94	$Ir^{3+} + 3\,e^- \rightleftharpoons Ir$	+1.156
Ru	–1.76	$Ru^{2+} + 2\,e^- \rightleftharpoons Ru$	+0.455
Os	–1.41	Os^{2+} unstable	N/A
Cd	–8.81	$Cd^{2+} + 2\,e^- \rightleftharpoons Cd$	−0.403
Zn	–7.25	$Zn^{2+} + 2\,e^- \rightleftharpoons Zn$	−0.762

Supplementary Table 3: Prediction errors as a function of the number of principal components: Mean absolute, standard, minimum, and maximum errors: MAE, σ, ε_-, and ε_+. All errors in eV. The arithmetic average of the errors is 0.00 eV for all values of $k_{\max}$, as the PCA includes a centering step. The eigenvalues of the covariance matrix, λ (in eV^2), are also included. The total variance is $\sum \lambda = 550.26$ eV^2, i.e., the trace of $\mathbf{D}$. Only 11 eigenvalues are non-zero due to the data centering (Supplementary Equation 4).

$k_{\max}$	λ	MAE	σ	ε_-	ε_+
1	509.96	0.153	0.218	–1.29	+1.00
2	30.07	0.081	0.109	–0.45	+0.50
3	3.62	0.065	0.089	–0.36	+0.31
4	1.84	0.057	0.076	–0.32	+0.29
⋮	⋮	⋮	⋮	⋮	⋮
11	0.12	0.000	0.000	–0.00	+0.00
12+	0.00	0.000	0.000	–0.00	+0.00

Supplementary Table 4: Benchmark of formation energies. The experimental coverage is shown for each reaction as measured in Ref. [15]. High-quality DFT data is provided from Ref. [16] for BEEF and PBE density functionals. The energies resulted from PCR-L1O, including zero-point energy corrections, are shown on column PCR. On transition metal surfaces, the vibrational frequencies of the adsorbates scale with their adsorption energies.[17] For CO adsorption at 0.25ML, we also considered repulsive adsorbate-adsorbate interactions from DFT to correct the adsorption energy, PCR_θ.

Surface	θ	Reaction	Exp.	BEEF[16]	PBE[16]	PCR	PCR_θ
Ni(111)	0.10	$CO \rightarrow {}^*CO$	–1.33	–1.51	–1.81	–1.68	–
Pt(111)	0.25	$CO \rightarrow {}^*CO$	–1.22	–1.68	–1.62	–2.17	–1.98
Pd(111)	0.25	$CO \rightarrow {}^*CO$	–1.44	–1.62	–1.80	–2.01	–1.84
Rh(111)	0.25	$CO \rightarrow {}^*CO$	–1.40	–1.68	–1.91	–2.04	–1.97
Ir(111)	0.25	$CO \rightarrow {}^*CO$	–1.61	–1.76	–1.96	–2.12	–2.09
Cu(111)	0.25	$CO \rightarrow {}^*CO$	–0.54	–0.57	–0.76	–0.88	–0.76
Ru(0001)	0.25	$CO \rightarrow {}^*CO$	–1.60	–1.67	–1.89	–2.15	–2.07
Ag(111)	0.25	$CO \rightarrow {}^*CO$	–0.23	–	–	–0.20	–0.08
Ni(111)	0.25	$O_2 \rightarrow 2O^*$	–4.93	–4.33	–4.60	–4.59	–
Pt(111)	0.11	$O_2 \rightarrow 2O^*$	–2.11	–1.97	–2.13	–2.39	–
Pt(111)	0.25	$H_2 \rightarrow 2H^*$	–0.73	–0.50	–0.82	–1.37	–
Ni(111)	0.25	$H_2 \rightarrow 2H^*$	–0.94	–0.66	–1.08	–0.70	–
Rh(111)	0.25	$H_2 \rightarrow 2H^*$	–0.69	–0.69	–1.16	–1.11	–
Pd(111)	0.25	$H_2 \rightarrow 2H^*$	–0.88	–0.69	–0.94	–1.13	–
Ir(111)	0.25	$D_2 \rightarrow 2D^*$	–0.59	–	–	–1.23	–
Ru(0001)	0.25	$H_2 \rightarrow 2H^*$	–1.17	–	–	–1.14	–
Pt(111)	0.11	$CH_3OH \rightarrow CH_3OH^*$	–0.58	–	–	–0.47	–
Pt(111)	0.67	$D_2O \rightarrow D_2O^*$	–0.52	–	–	–0.35	–
Pt(111)	0.50	$CH_4 \rightarrow CH_4^*$	–0.16	–	–	–0.09	–
Pt(111)	0.33	$C_2H_6 \rightarrow C_2H_6$	–0.29	–	–	–0.22	–
Pt(111)	–	$C_2H_4 \rightarrow {}^{**}CH–CH_3+H^*$	–1.41	–	–	–1.13	–
Pt(111)	0.17	$C_2H_4 \rightarrow {}^{3*}C–CH_3+\frac{1}{2}H_2$	–1.01	–	–	–1.54	–

Supplementary Table 5: Labels for the species used in this study. The choosen species are taken from our previous studies on Cu, Ru, Pd, and Pt.[6,18]

Label	Species
0000	Clean surface
0101	H*
0011	O*
0111	OH*
0211	H_2O*
1001	C*
1101	CH*
1201	CH_2*
1301	CH_3*
1401	CH_4*
1011	CO*
1111	CHO*
1112	COH*
1211	CH_2O*
1212	CHOH*
1311	CH_3O*
1312	CH_2OH*
1411	CH_3OH*
2001	C_2*
2101	C_2H*
2201	CH_2C*
2202	CHCH*
2301	CH_3C*
2302	CH_2CH*
2401	CH_3CH*
2402	CH_2CH_2*
2501	CH_3CH_2*
2601	CH_3CH_3*
2011	CCO*
2111	CHCO*
2112	CCHO*
2113	CCOH*
2211	CH_2CO*
2212	CHCHO*
2213	CCH_2O*
2214	CHCOH*
2215	CCHOH*
2311	CH_3CO*
2312	CH_2CHO*
2313	$CHCH_2O$*

Continues on next page

Supplementary Table 5

Label	Species
2314	CH_2COH^*
2315	$CHCHOH^*$
2316	CCH_2OH^*
2411	CH_3CHO^*
2412	$CH_2CH_2O^*$
2413	CH_3COH^*
2414	CH_2CHOH^*
2415	$CHCH_2OH^*$
2511	$CH_3CH_2O^*$
2512	CH_3CHOH^*
2513	$CH_2CH_2OH^*$
2611	$CH_3CH_2OH^*$
2121	$OCCHO^*$
2122	$OCCOH^*$
2221	$OCHCHO^*$
2222	$OCCH_2O^*$
2223	$HOCCHO^*$
2224	$HOCHCO^*$
2225	$HOCCOH^*$
2321	OCH_2CHO^*
2322	$HOCCH_2O^*$
2323	$HOCHCHO^*$
2324	$OHCH_2CO^*$
2325	$HOCHCOH^*$
2421	$OCH_2CH_2O^*$
2422	$HOCHCH_2O^*$
2423	$HOCH_2CHO^*$
2424	$HOCH_2COH^*$
2425	$HOCHCHOH^*$
2521	$HOCH_2CH_2O^*$
2522	$HOCH_2CHOH^*$
2621	$HOCH_2CH_2OH^*$

Supplementary Figures

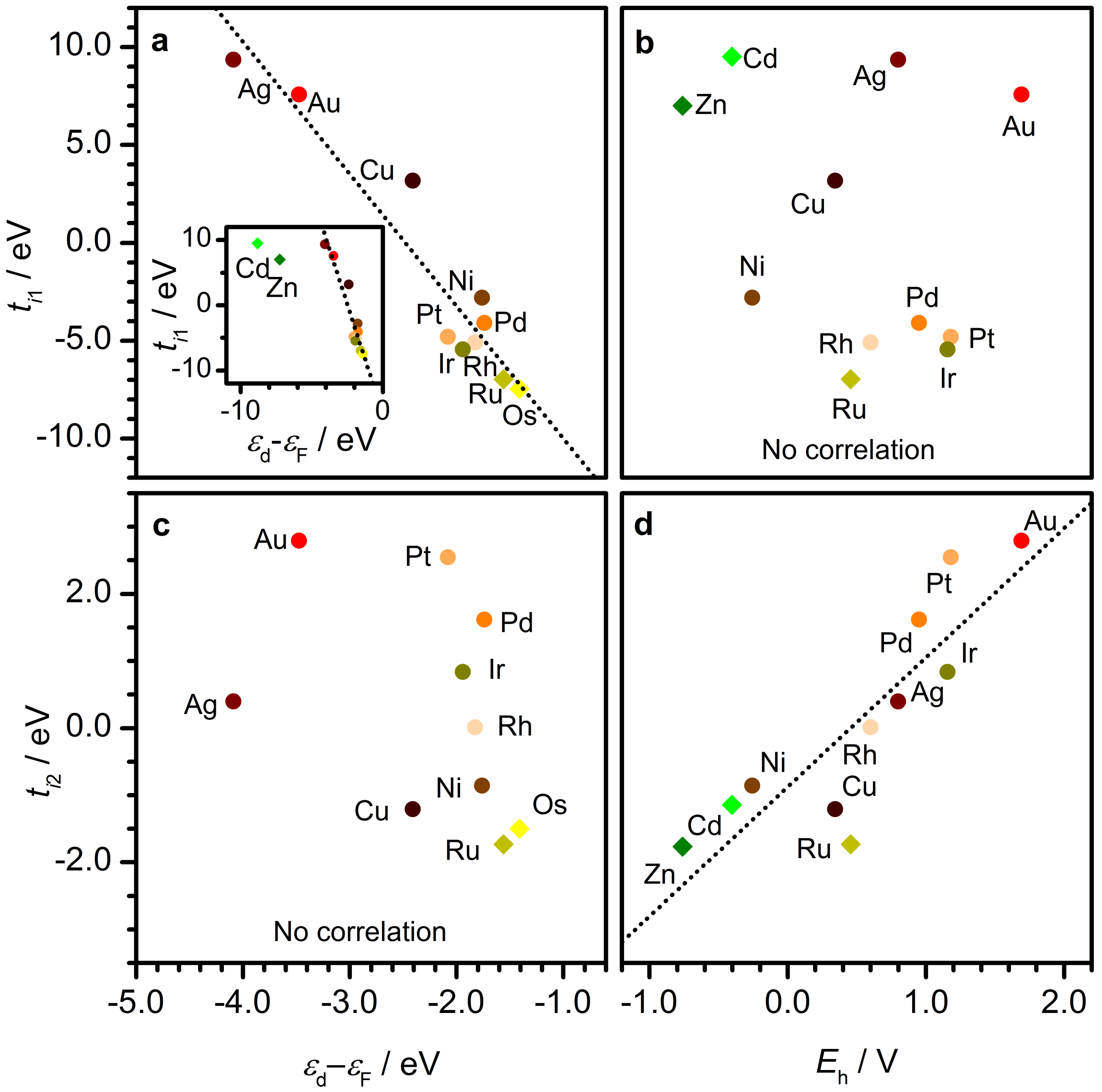

Supplementary Figure 1: First and second descriptors for the metal, t_{i1} and t_{i2}, plotted against the d-band centers (**a,c**) and the reduction potentials[7] (**b,d**). The d-band center modulates the adsorption strength on late transition metals (groups 8-11),[19,20] but it cannot describe the behavior of Zn and Cd.

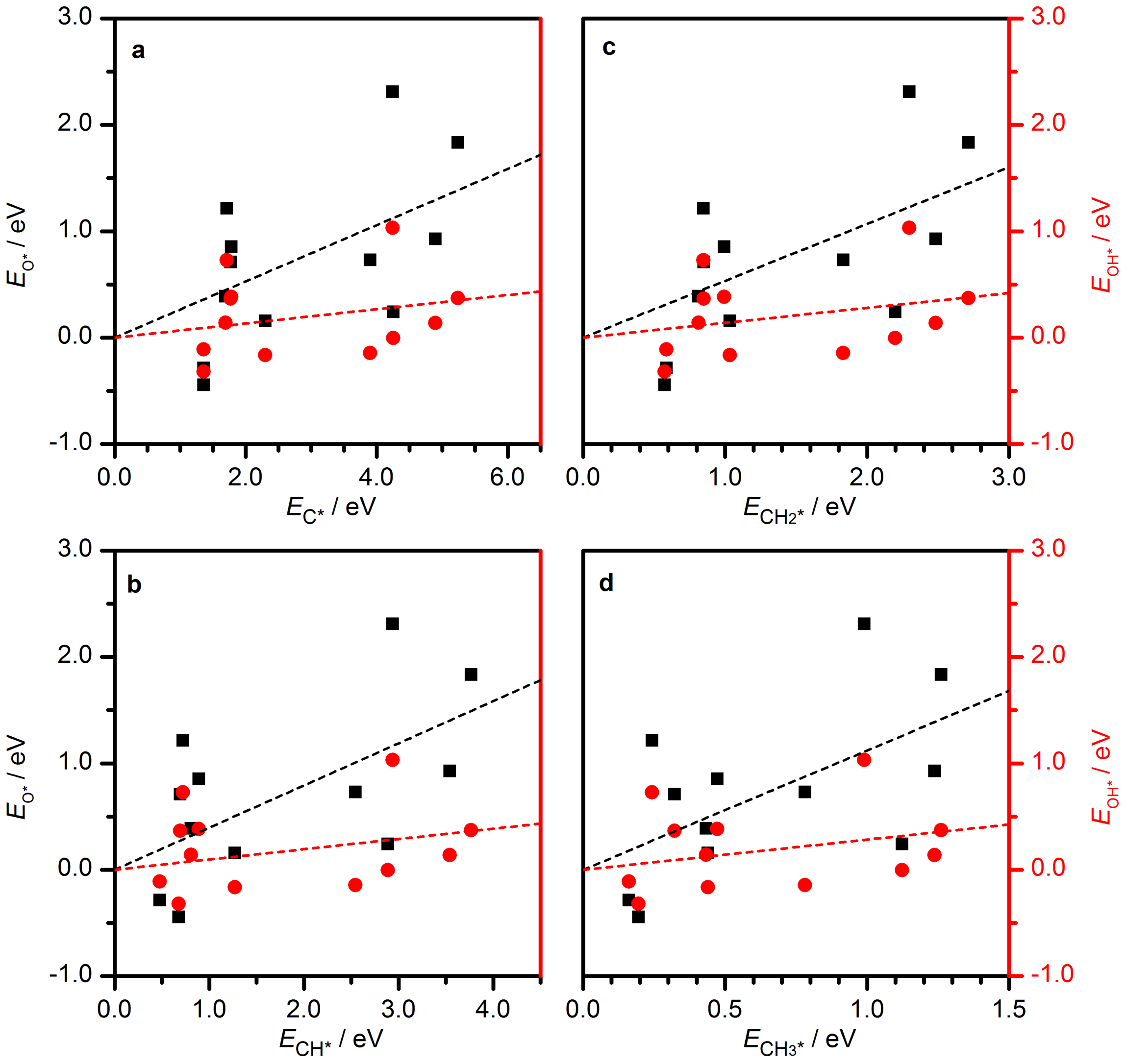

Supplementary Figure 2: Formation energies of O* (black) or OH* (red) plotted against **a** C, **b** CH, **c** CH_3*, and **d** CH_3*.

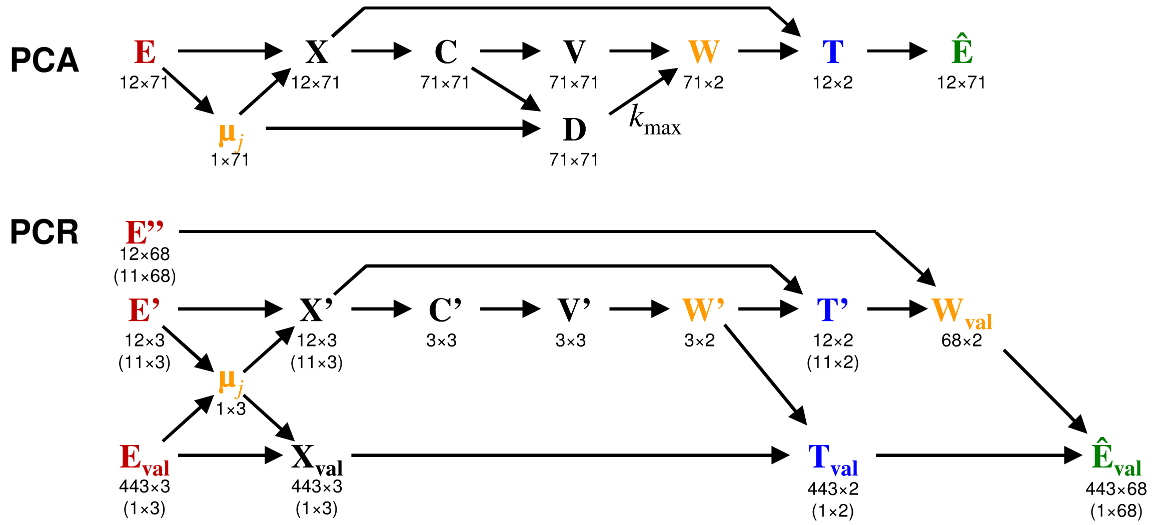

Supplementary Figure 3: Data flow diagram for PCA and PCR. The formation energy of species j on metal i is obtained from DFT and Equations 1-2. All the energies are grouped in thermochemistry matrices, in red. The approximations done *via* PCA/PCR are shown in green. Variables associated with metals and species are shown in blue and orange, respectively. Variables associated with mathematical procedures are in black. The size of the matrices used in this study are shown below. Those in parentheses corresponds to the PCR-L1O procedure when they were different from the PCR applied on single-atom and near-surface alloys.

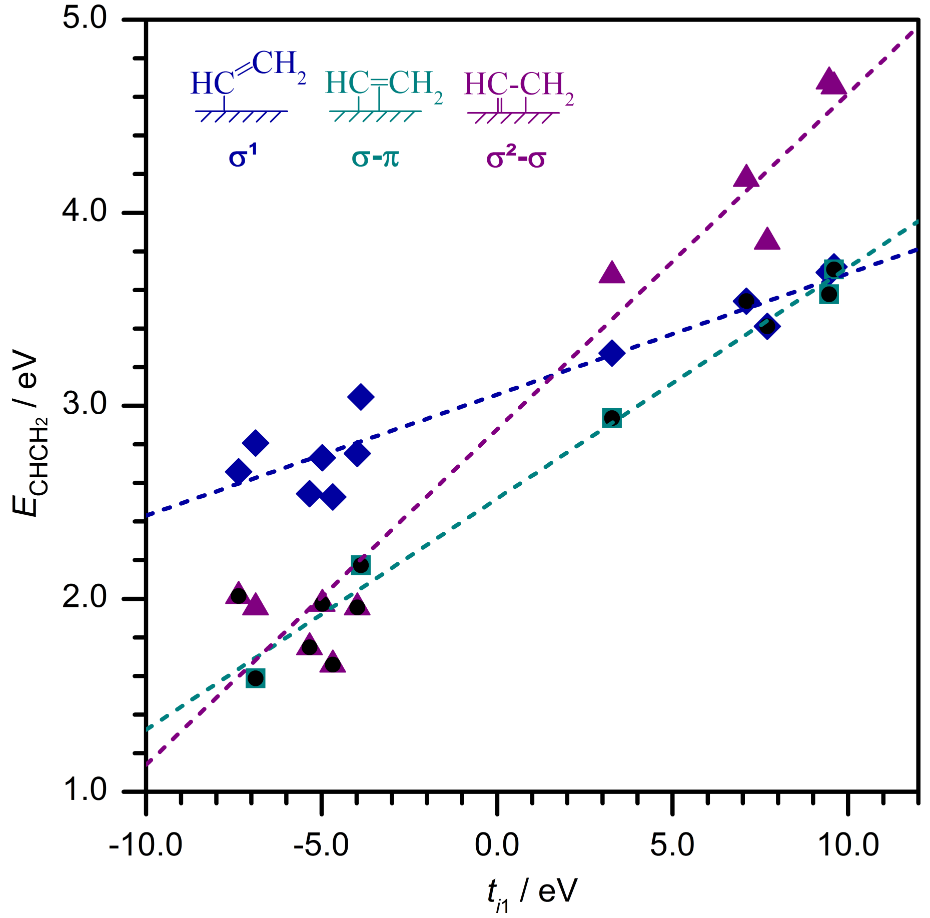

Supplementary Figure 4: Formation energy of three conformations of $CHCH_2$ plotted against the first metal descriptor, t_{i1}: σ^1 (blue diamonds), σ-π (green squares), and σ^1-σ^2 (purple triangles). The most stable conformations are marked as black circles.

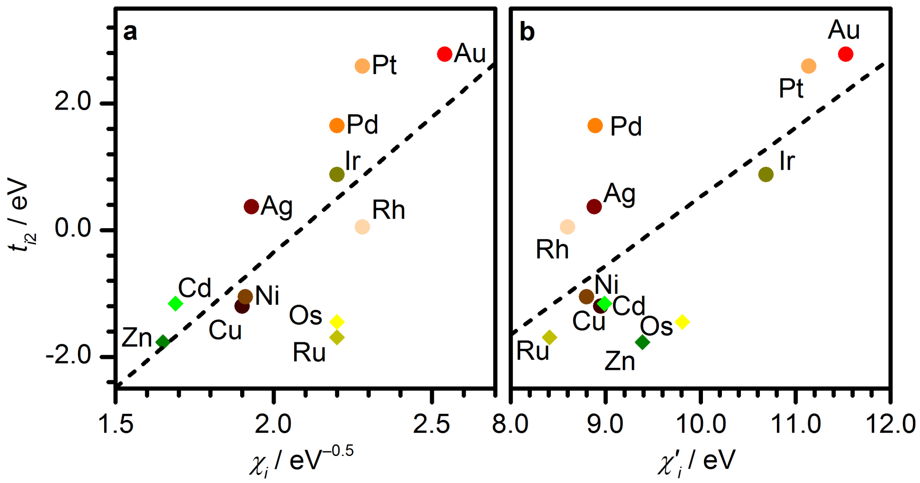

Supplementary Figure 5: Second descriptor for the metals, t_{i2}, plotted against **a** Pauling and **b** Mulliken electronegativity.[7] Pauling's electronegativity scale, χ_i in $eV^{-0.5}$, is defined from valence bond theory as the additional stabilization of a heteronuclear bond due to ionic contribution. Mulliken's electronegativity, χ'_i in eV, is the average of electron affinity and ionization potential, which are defined for isolated neutral atoms in gas phase, instead of bulk metals.

Supplementary References

[1] James, G., Witten, D., Hastie, T. & Tibshirani, R. *An introduction to statistical learning* (Springer series in statistics, 2013).

[2] Rothenberg, G. Data mining in catalysis: Separating knowledge from garbage. *Catal. Today* **137**, 2–10 (2008).

[3] Jones, G., Jakobsen, J. G., Shim, S. S., Kleis, J., Andersson, M. P., Rossmeisl, J., Abild-Pedersen, F., Bligaard, T., Helveg, S., Hinnemann, B., Rostrup-Nielsen, J. R., Chorkendorff, I., Sehested, J. & Nørskov, J. K. First principles calculations and experimental insight into methane steam reforming over transition metal catalysts. *J. Catal.* **259**, 147–160 (2008).

[4] Lausche, A. C., Medford, A. J., Khan, T. S., Xu, Y., Bligaard, T., Abild-Pedersen, F., Nørskov, J. K. & Studt, F. On the effect of coverage-dependent adsorbate–adsorbate interactions for CO methanation on transition metal surfaces. *J. Catal.* **307**, 275–282 (2013).

[5] Greeley, J. & Mavrikakis, M. Alloy catalysts designed from first principles. *Nat. Mater.* **3**, 810–815 (2004).

[6] Li, Q., García-Muelas, R. & López, N. Microkinetics of alcohol reforming for H_2 production from a FAIR density functional theory database. *Nat. Commun.* **9**, 526 (2018).

[7] Lide, D. *CRC Handbook of Chemistry and Physics* (CRC press LLC, 2003–2004), 84th edn.

[8] Grimme, S. Semiempirical GGA-type density functional constructed with a long-range dispersion correction. *J. Comput. Chem.* **27**, 1787–1799 (2006).

[9] Almora-Barrios, N., Carchini, G., Błoński, P. & López, N. Costless derivation of dispersion coefficients for metal surfaces. *J. Chem. Theory Comput.* **10**, 5002–5009 (2014).

[10] Kresse, G. & Furthmüller, J. Efficiency of ab-initio total energy calculations for metals and semiconductors using a plane-wave basis set. *Comput. Mater. Sci.* **6**, 15–50 (1996).

[11] Kresse, G. & Furthmüller, J. Efficient iterative schemes for ab initio total-energy calculations using a plane-wave basis set. *Phys. Rev. B* **54**, 11169–11186 (1996).

[12] Perdew, J. P., Burke, K. & Ernzerhof, M. Generalized gradient approximation made simple. *Phys. Rev. Lett.* **77**, 3865–3868 (1996).

[13] Blöchl, P. E. Projector augmented-wave method. *Phys. Rev. B* **50**, 17953–17979 (1994).

[14] Kresse, G. & Joubert, D. From ultrasoft pseudopotentials to the projector augmented-wave method. *Phys. Rev. B* **59**, 1758–1775 (1999).

[15] Silbaugh, T. L. & Campbell, C. T. Energies of formation reactions measured for adsorbates on late transition metal surfaces. *J. Phys. Chem. C* **120**, 25161–25172 (2016).

[16] Wellendorff, J., Silbaugh, T. L., García-Pintos, D., Nørskov, J. K., Bligaard, T., Studt, F. & Campbell, C. T. A benchmark database for adsorption bond energies to transition metal surfaces and comparison to selected DFT functionals. *Surf. Sci.* **640**, 36–44 (2015).

[17] Lansford, J. L., Mironenko, A. V. & Vlachos, D. G. Scaling relationships and theory for vibrational frequencies of adsorbates on transition metal surfaces. *Nat. Commun.* **8**, 1842 (2017).

[18] García-Muelas, R., Li, Q. & López, N. Density functional theory comparison of methanol decomposition and reverse reactions on metal surfaces. *ACS Catal.* **5**, 1027–1036 (2015).

[19] Hammer, B. & Nørskov, J. K. Why gold is the noblest of all the metals. *Nature* **376**, 238–240 (1995).

[20] Hammer, B., Morikawa, Y. & Nørskov, J. K. CO chemisorption at metal surfaces and overlayers. *Phys. Rev. Lett.* **76**, 2141 (1996).
